# PP41 Enhancing Health Technology Assessment Through Behavioral Economics: A Strategic Approach To Sustainable Healthcare Innovation

**DOI:** 10.1017/S0266462324002137

**Published:** 2025-01-07

**Authors:** Shilpi Swami

## Abstract

**Introduction:**

Behavioral economics (BE) offers a spectrum of underutilized tools that could significantly enhance health technology assessment (HTA). This study proposes integration of BE into HTA, recognizing that understanding behavioral drivers can help in evaluating health technologies’ sustainability and adoption rates, thereby aligning HTA more closely with real-world healthcare dynamics and patient needs.

**Methods:**

The study reviews existing key BE concepts and proposals for integrating BE concepts such as loss aversion, present bias, framing effects, and social norms influence into HTA framework. Loss aversion refers to the tendency of people to prefer avoiding losses rather than acquiring equivalent gains, while present bias is the tendency to prioritize immediate rewards over future benefits. Framing effect is the cognitive bias where people decide on options based on whether they are presented with positive or negative connotations.

**Results:**

Loss aversion can increase patient adherence to chronic disease management technologies by emphasizing the avoidance of negative health outcomes. Present bias limits adopting preventive technologies, where immediate costs overshadow long-term benefits. Framing effects may determine how the presentation of technologies influences acceptance; for example, by framing a new surgical device as reducing the risk of postoperative complications (a positive frame), rather than not increasing the risk (a negative frame), it may be more readily accepted. Additionally, use of default options in electronic health records can improve data accuracy, and recognizing social norms can drive broader adoption and success of telehealth solutions.

**Conclusions:**

Integrating BE with HTA methodology offers a pathway to more patient-centered and sustainable health technology implementation, with an emphasis on patient behavior patterns. This approach enables HTA to more effectively address human behavior intricacies, ensuring that health technology evaluations are both comprehensive and relevant to the evolving needs of healthcare systems and patients.

